# RETRACTED ARTICLE: Medical history predicts phenome-wide disease onset and enables the rapid response to emerging health threats

**DOI:** 10.1038/s41467-024-48568-8

**Published:** 2024-05-20

**Authors:** Jakob Steinfeldt, Benjamin Wild, Thore Buergel, Maik Pietzner, Julius Upmeier zu Belzen, Andre Vauvelle, Stefan Hegselmann, Spiros Denaxas, Harry Hemingway, Claudia Langenberg, Ulf Landmesser, John Deanfield, Roland Eils

**Affiliations:** 1 Department of Cardiology, Angiology and Intensive Care Medicine, Deutsches Herzzentrum der Charité (DHZC), Berlin, Germany; 2 Charité – Universitätsmedizin Berlin, corporate member of Freie Universität Berlin and Humboldt Universität zu Berlin, Klinik/Centrum, Charitéplatz 1, 10117 Berlin, Germany; 3 Computational Medicine, Berlin Institute of Health (BIH), Charite - University Medicine Berlin, Berlin, Germany; 4 Friede Springer Cardiovascular Prevention Center@Charite, Charite - University Medicine Berlin, Berlin, Germany; 5 Institute of Cardiovascular Sciences, University College London, London, UK; 6 Center for Digital Health, Berlin Institute of Health (BIH), Charite - University Medicine Berlin, Berlin, Germany; 7 MRC Epidemiology Unit, Institute of Metabolic Science, University of Cambridge, Cambridge, UK; 8 Precision Health University Research Institute, Queen Mary University of London and Barts NHS Trust, London, UK; 9 Institute of Health Informatics, University College London, London, UK; 10 Institute for Medical Engineering and Science, Massachusetts Institute of Technology, Massachusetts, USA; 11 Pattern Recognition and Image Analysis Lab, University of Münster, Münster, Germany; 12 British Heart Foundation Data Science Centre, London, UK; 13 Health Data Research UK, London, UK; 14 National Institute for Health Research, Biomedical Research Centre at University College London Hospitals National Institute for Health Research, Biomedical Research Centre, London, UK; 15 Berlin Institute of Health (BIH), Charite - University Medicine Berlin, Berlin, Germany; 16 DZHK (German Centre for Cardiovascular Research), Partner Site Berlin, Berlin Berlin, Germany; 17 Health Data Science Unit, Heidelberg University Hospital and BioQuant, Heidelberg, Germany

**Keywords:** Disease prevention, Statistics, Epidemiology

The authors have retracted this article at the request of the UK Biobank. They had used patient data from the UK Biobank study to assess risk stratification in a range of diseases; however, according to the Control of Patient Information (COPI) notice, primary care data had only been made available for research directly related to COVID-19. Therefore, this article has been retracted and the content removed.

All authors agree with this retraction.

A revised article [1] reports the analyses with all patient data removed that should not be used according to the Control of Patient Information (COPI) notice. It has undergone peer review independently from the original article’s review process and has been published.

**REFERENCES**

1. Steinfeldt, J., Wild, B., Buergel, T. et al. Medical history predicts phenome-wide disease onset and enables the rapid response to emerging health threats. *Nat Commun* **16**, 585 10.1038/s41467-025-55879-x (2025).

